# Impaired Replication Timing Promotes Tissue-Specific Expression of Common Fragile Sites

**DOI:** 10.3390/genes11030326

**Published:** 2020-03-19

**Authors:** Klizia Maccaroni, Elisa Balzano, Federica Mirimao, Simona Giunta, Franca Pelliccia

**Affiliations:** 1 Dipartimento di Biologia e Biotecnologie “Charles Darwin”, Sapienza Università di Roma, 00185 Roma, Italy; Klizia.Maccaroni@uniroma1.it (K.M.); Elisa.Balzano@uniroma1.it (E.B.); federicamirimao@gmail.com (F.M.); 2 The Rockefeller University, 1230 York Avenue, New York, NY 10065, USA

**Keywords:** common fragile sites, DNA replication timing, replicative stress, tissue specificity, human fetal fibroblasts, chromosomal instability, fluorescent in situ hybridization, gene length, long genes

## Abstract

Common fragile sites (CFSs) are particularly vulnerable regions of the genome that become visible as breaks, gaps, or constrictions on metaphase chromosomes when cells are under replicative stress. Impairment in DNA replication, late replication timing, enrichment of A/T nucleotides that tend to form secondary structures, the paucity of active or inducible replication origins, the generation of R-loops, and the collision between replication and transcription machineries on particularly long genes are some of the reported characteristics of CFSs that may contribute to their tissue-specific fragility. Here, we validated the induction of two CFSs previously found in the human fetal lung fibroblast line, Medical Research Council **cell** strain **5** (MRC-5), in another cell line derived from the same fetal tissue, Institute for Medical Research-90 cells (IMR-90). After induction of CFSs through aphidicolin, we confirmed the expression of the CFS 1p31.1 on chromosome 1 and CFS 3q13.3 on chromosome 3 in both fetal lines. Interestingly, these sites were found to not be fragile in lymphocytes, suggesting a role for epigenetic or transcriptional programs for this tissue specificity. Both these sites contained late-replicating genes NEGR1 (neuronal growth regulator 1) at 1p31.1 and LSAMP (limbic system-associated membrane protein) at 3q13.3, which are much longer, 0.880 and 1.4 Mb, respectively, than the average gene length. Given the established connection between long genes and CFS, we compiled information from the literature on all previously identified CFSs expressed in fibroblasts and lymphocytes in response to aphidicolin, including the size of the genes contained in each fragile region. Our comprehensive analysis confirmed that the genes found within CFSs are longer than the average human gene; interestingly, the two longest genes in the human genome are found within CFSs: Contactin Associated Protein 2 gene (*CNTNAP2*) in a lymphocytes’ CFS, and Duchenne muscular dystrophy gene (*DMD)* in a CFS expressed in both lymphocytes and fibroblasts. This indicates that the presence of very long genes is a unifying feature of all CFSs. We also obtained replication profiles of the 1p31.1 and 3q13.3 sites under both perturbed and unperturbed conditions using a combination of fluorescent in situ hybridization (FISH) and immunofluorescence against bromodeoxyuridine (BrdU) on interphase nuclei. Our analysis of the replication dynamics of these CFSs showed that, compared to lymphocytes where these regions are non-fragile, fibroblasts display incomplete replication of the fragile alleles, even in the absence of exogenous replication stress. Our data point to the existence of intrinsic features, in addition to the presence of long genes, which affect DNA replication of the CFSs in fibroblasts, thus promoting chromosomal instability in a tissue-specific manner.

## 1. Introduction

Common fragile sites (CFSs) are regions in which the DNA is prone to gaps, breakage, or constriction that can be visualized on metaphase chromosomes when cells are under replicative stress [1]; they represent about 1% of the whole genome [2], spanning from hundreds to thousands of kilobases in size. The frequency of sister chromatid exchange is also higher at CFSs compared to non-fragile regions. Interestingly, they represent sites of exogenous viral DNA integration. Altogether, these features make CFSs extremely recombinogenic and genetically unstable regions of the human genome [3,4].

Despite these destabilizing characteristics, CFSs can be found in almost every organism, from prokaryotes to eukaryotes, suggesting a positive evolutionary selection of these loci [5].

CFSs are found in all individuals of a population as part of chromosomes’ normal structure; they are categorized by differences in frequency [6], and they are expressed when cells are cultured under conditions of replicative stress, such as aphidicolin (APH), a compound that acts directly on enzymes responsible for DNA replication [3]. Perhaps unsurprisingly, several CFSs can co-localize with one or more genes in the genome. For instance, FRAgile site 3B (FRA3B) and FRA16D CFSs co-localize with two tumor suppressor genes, the fragile histidine triad (*FHIT*) gene and the WW domain-containing oxidoreductase (*WWOX*), respectively [7,8], two genes known for their involvement in chromosomal aberrations and found mutated in different type of tumors [9,10].

Historically speaking, pioneering studies on CFSs have been made in human lymphocytes, mostly because of the simplicity in eliciting their fragility using APH in this cell type. Importantly, however, it has been found that CFS expression differs across cell lines [11]. The identification of CFSs unique to fibroblasts, epithelial colon, breast cell lines, and erythrocytes cell lines [12,13] added a layer of complexity to CFSs classification and expression. Using genome-wide approaches such as Repli-seq combined with classic cytogenetic techniques, Le Tallec et al. [14] identified new CFSs in human fibroblast cell lines that differ from the ones localized on human lymphocytes. Comparing replication timing of the CFSs in fibroblasts and lymphocytes, key difference have emerged—in lymphocytes, the core (the most fragile region in a CFS) of FRA3B is replicated early in S-phase from flanking origins where the replisome must cover long distances; in fibroblasts instead, the same core region is replicated during late S-phase and often continues into G2. This raises the possibility that different number of DNA replication origins can be activated for the same region in different cell types [15], a phenomenon likely to be epigenetically specified [6]. 

CFSs are expressed when cells are under replicative stress, connecting their fragility to replication. Delayed completion of region duplication during metaphase can cause missegregation of underreplicated DNA and breaks during mitosis. In mammals, perturbations in replication fork progression can also stochastically alter the replication rate from one region to another. This is due to the complexity of eukaryotic genomes both in size and structure, and these factors can affect replication due to slower fork progression or paused/stalled replisomes that are more prone to collapsing [16,17]. This is confirmed by APH acting to significantly slow the replication of the most expressed CFSs (FRA3B) in lymphocytes, where 16.5% of the fragile region remains unreplicated upon entering G2 phase [18]. 

Low doses of APH act to slow the polymerase, hence the replication rate, while the helicase-topoisomerase I complex gets uncoupled and continues to unwind DNA, leaving long regions of exposed unreplicated single-strand DNA (ssDNA) that activates the checkpoint [19]. The fragile regions analyzed by FlexStab software show that many flexible regions are present within CFSs, named “flexibility peaks” [20,21]. It has been demonstrated that these “flexible sequences” are enriched in adenine-thymine rich (AT-rich) repetitive DNA that can form hairpins or cruciform secondary structures that could represent barriers to replicative fork progression, promoting genomic instability and fragile site expression [22], resulting in the activation of checkpoint mechanisms that block mitosis entry. 

In mammals, to replicate correctly and completely all the genomic material within the time of S-phase, at least 30,000 to 50,000 replication origins should be activated; this value exceeds the number of origins effectively activated during DNA replication, inferring that changes in replication fork speed may play a role in ensuring completion of genome duplication within the required timeframe [23].

Furthermore, several CFSs contain long genes [24]. Interestingly, these long genes have common molecular characteristics—they are often late replicating and AT-rich [25], which are also true for the entire CFS region. It has been demonstrated that transcription of some particularly long genes found within CFSs could take more than one cell cycle to be completed, inevitably leading to simultaneous transcription and replication. Head-on collision between the two molecular machineries is able to cause DNA breaks. That is why replication and transcription are co-directional, especially for essential genes, or found as temporally separated events in certain eukaryotes [26]. Yet, this begs the question as to why such long genes have evolved, considering the challenges posed to genome stability. 

Given the previously reported connection between the tissue-specific fragility of CFSs, presence of long genes, and replication timing, here we set out to study gene content and replication dynamics in two human fetal lung fibroblast lines, the previously characterized MRC-5 and a novel line, IMR-90, comparing them against lymphocytes where these regions are not fragile. We found that CFS expression in the novel line we characterized, IMR-90, followed a different pattern of replication to that found in MRC-5, yet both displayed unfinished replication of the CFS by late S-phase. Interestingly, we found that replication remained incomplete in presence of APH, yet both fibroblast lines showed a delay in completion of replication compared to lymphocytes even in unperturbed conditions. Furthermore, we provided for the first time a comprehensive representation of all genes contained within CFS expressed in fibroblasts and lymphocytes, highlighting a clear association between gene length and loci fragility. In agreement with previous work, our direct characterization of replication and expression of CFS comparing fibroblast lines from the same tissue types to lymphocytes strengthens the association between delayed/unfinished replication, above average gene length, and fragility of the region.

## 2. Materials and Methods 

### 2.1. Human Cell Lines and CFS Induction

Peripheral blood lymphocytes were taken from two unrelated, healthy individuals (one female, one male) with normal karyotype who agreed to participate by written informed consent. Lymphocytes were grown in Roswell Park Memorial Institute (RPMI) 1640 medium (Corning) with 10% fetal bovine serum (FBS, Corning) and 0.15 mL of phytohemagglutinin (GIBCO) for 72 hours before. MRC-5 and IMR-90, two human fetal lung fibroblast cell lines of 14 weeks gestation and 16 weeks gestation, respectively, were grown in Minimum Essential Medium (MEM) 1X medium (GIBCO) supplemented with 10% FBS (Corning). All media were supplemented with 1% L-glutamine (Corning) and 1X penicillin/streptomycin, and the cells were incubated at 37°C with 5% CO_2_. 

For CFS induction, all cell lines were treated with APH. Lymphocytes were treated with 0.4 μM of APH (Sigma-Aldrich) for 24 hours and 10 mM colchicine (Sigma-Aldrich) for 2 hours. Both fibroblasts cell lines were treated with 0.3 μM of APH for 22 hours and 10 mM colchicine (Sigma-Aldrich) for 4 hours prior collecting the cells. 

### 2.2. Metaphase Spread Preparation

Lymphocytes were centrifuged at 2000 rpm for 5 min prior to adding hypotonic solution (KCl 75 mM) for 6 min. After centrifugation, Ibraimov solution (5% acetic acid, 3% methanol in distilled H_2_O) was added as a pre-fixation step. The pellet was washed twice with −20°C fixative (methanol/acetic acid—3:1) and stored overnight at 4 °C.

After detachment with 0.05% trypsin (Corning), fibroblasts were centrifuged for 5 min at 1200 rpm and the pellet was resuspended in 5 ml of pre-warmed (37 °C) 75 mM KCl hypotonic solution (Sigma) for 6 min at room temperature (RT). After two centrifugations at 1200 rpm for 5 min, swollen cells were resuspended with cold (−20 °C) fixative (methanol/acetic acid—3:1) twice. Fixed cells were stored at −20 °C overnight. 

Cell suspension involved dropping on clean, wet slides, which were then air dried overnight; the slides were stored in the dark at 4 °C.

### 2.3. R-banding with Chromomycin A_3_

Metaphase slides were washed for 10 min with phosphate buffer before chromomycin A_3_ (CMA3) solution (10 mg/ml) addition (Sigma) and left for 2 hours at RT in a dark, humified chamber. The slides were then rinsed in NaCl-HEPES (4-(2-Hydroxyethyl)piperazine-1-ethanesulfonic acid) buffer and stained for 15 min in methyl green solution (Sigma). After two washes in NaCl-HEPES buffer, the antifading (Vectashield H-1200/isopropilgallate—1:300; Vector Laboratories, Burlingame, CA) was added. The slides were stored at 4 °C for 3 days in the dark, prior observation on a fluorescent Zeiss Axioplan microscope with CCD.

### 2.4. BAC Extraction and Labelling by Nick Translation

Two bacterial artificial chromosomes (BAC) were chosen from GenBank [27] for each putative fragile region on chromosome 1 and 3 (RP11-316C12, chr1: 71,385,313-71,476,945; RP11-297N6 chr1: 7,346,775-7,544,907; RP11-324H4 chr3: 116,954,325-117,125,019; RP11-305I9 chr3: 119,265,025-119,399,070).

Bacterial cells were grown in 10 mL of Luria-Bertani (LB) medium with the addition of 20 μg/mL chloramphenicol. The extraction was carried out by alkaline lysis protocol.

The BAC extracted were labelled by nick translation with Deoxy-Uridine Triphosphate-16-UTP (bio-16-dUTP) and/or Digoxigenin-16-dUTP (dig-16-dUTP) (Roche) and used as probes for fluorescent in situ hybridization (FISH) experiments on metaphases or interphase nuclei. 

### 2.5. Fluorescence In Situ Hybridization 

The slides were treated with 100 μg/mL RNase solution (Thermo Scientific) for one hour at 37 °C in a humified chamber and successively dehydrated with 70%, 90%, and 100% ethanol solution for 5 min each and air dried. Afterwards, the slides were aged for 1 hour at 65 °C. DNA was denatured at 80 °C for 2 min with 70% formamide (Sigma) 2 × SSC. (Saline-Sodium Citrate) The denaturation was blocked with cold (−20 °C) 70% ethanol and dehydrated with 90% and 100% ethanol washes and air dried. A total of 200 ng of probe was used for each slide and was denatured at 80 °C for 8 min, and then incubated for 15 min at 37 °C. Slides were incubated with denatured probe overnight at 37 °C.

Post-hybridization washes were performed three times in 1 × SSC at 60 °C, and the slides were labelled with antibody anti-digoxigenin-rhodamine (1:300, Roche), Fluorescein-5-isothiocyanate (FITC) anti-digoxigenin-rhodamine (1:20 Roche), Cy-3 streptavidin (1:300), or FITC-avidin (1:20). Three washes in 2 × SSC 0.1% Tween20 were performed, and the slides were counterstained with 1:300 DAPI (4′,6′-diamidino-2-phenylindole hydrochloride, 1 μg/mL; Sigma) and mounted in Vectashield medium (Vector Laboratories, Burlingame).

Slides were observed with a fluorescence microscope (Zeiss Axioplan) equipped with a CCD (Charge-Coupled Device) camera for image capturing. Greyscale images for fluorophore and DAPI signals were acquired separately, pseudo-colored, and merged using the Photoshop software.

### 2.6. Immunofluorescence against Bromodeoxyuridine

To identify replicating interphase nuclei, 10 μM of bromodeoxyuridine was added to the culture medium 20 min prior harvesting the cells. The same slides treated with FISH probes were washed once with 4 × SSC 0.1% Tween20 (Sigma-Aldrich) solution, followed by 2 hours of incubation with primary antibody anti-bromodeoxyuridine (BrdU) (1:1000, Thermo-Fisher). After three washes in PBS 1X (Phosphate Buffered Saline 1X), the secondary antibody FITC-anti-mouse IgG (Immunoglobulin G) (1:100, Thermo-Fisher) was added for 1 hour. The slides were washed four times in PBS 1× and mounted with Vectashield H-1200 with DAPI (Vector Laboratories, Burlingame, CA). The slides were stored at 4 °C overnight before observation on a fluorescent Zeiss Axioplan microscope equipped with CCD. The quantification shown in the histograms (Figures 5 and 6) display the mean and the standard error of the mean (SEM) from three independent experiments with more than 30 nuclei in each stage (early, mid-, late-S-phase). The *p*-value was calculated by comparing each time-point and experimental condition for IMR-90 and MRC-5 against the correspondent time-point and treatment in lymphocytes using a two-tailed *t*-test. The *p*-values represented correspond to non-significant (ns) *p* > 0.05, * for *p* ≤ 0.05, ** for *p* ≤ 0.01, and *** for *p* ≤ 0.001. Trends of replication dynamics (Figures S5–S7) were obtained by qualitative BrdU classification into five temporal stages: early (phase I; pan-nuclear BrdU), between early and mid (phase II; patchy pan-nuclear BrdU staining), mid (phase III; BrdU staining in ≈50% of nuclear area), between mid and late (phase IV; BrdU speckles covering ≈30% of the nucleus), and late (phase V; few remaining speckles of BrdU signal) S-phase (Figure S5). We plotted the percentage of replicated alleles, counting at least 30 nuclei for each phase for all 3 lines in both unperturbed and stress conditions.

### 2.7. Gene Length Analysis

Studies describing CFS expression in fibroblasts (in blue) and in lymphocytes (in black) were pooled and annotated in a separate an Excel spreadsheet (Table 1). The span of the fragile regions was identified and the complete annotation of genes and transcribed regions within the region, excluding non-coding RNAs, were annotated (Table 1), including the size (Mb) for each derived from National Center for Biotechnology Information (NCBI [28]); search updated to January 2020) and verified from Ensembl Genome Browser [29] where needed. Length in base pair (bp) of all protein-coding genes was plotted and analyzed using Prism 5.0 (GraphPad Software). 

### 2.8. DNA Repeat Composition Analysis 

The presence of repetitive DNA and other genomic elements was analyzed using the NCBI public database [30]. The resulting DNA repeats and sequence composition for the fragile region was compared against non-fragile loci (N-FRA) [31] and a human genome average (HGA) [32] with a matched AT content to the CFS 1p31.1 (64%) and 3q13.3 (63%).

## 3. Results

### 3.1. Cytogenetic Localization of Fragile Sites

**Figure 1:**
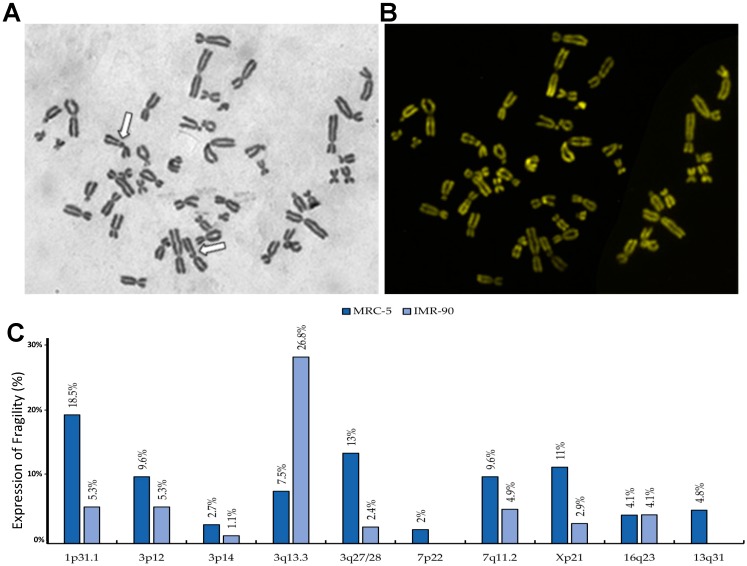
Cytogenetic localization and expression of common fragile sites in fibroblast cell lines. (**A**) IMR-90 metaphase stained with Giemsa. The white arrow shows the break on chromosomes 3 (3q13.3). (**B**) The same sample stained with CMA3 for more precise localization by banding. (**C**) The histogram showing the percentage of breaks expressed in both MRC-5 and IMR-90 cell lines as an average of three experiments; the breaks with frequency lower than 3% were not considered, given their uncommon fragility.

To assign the regions of fragility to specific loci, we induced CFSs in both MRC-5 and IMR-90 fibroblast cell lines cultured with medium with the addition of APH to induce replicative stress. Chromosome breaks were identified with Giemsa staining, and more precisely localized cytogenetically using R-banding (Figure 1A,B). The break sites were found in fibroblasts but absent in lymphocytes.

To be classified as a bona fide CFS, a break must be found with a frequency higher than 3%. In our fibroblast cell lines, we found the frequency of their expression under our experimental conditions to be 18.5% for 1p31.1 located on chromosome 1 in MRC-5 and a 5.3% frequency of expression in IMR-90; 3q13.3 located on chromosome 3 had a 7.5% frequency of expression in MRC-5 and 26.8% frequency of expression in IMR-90 (Figure 1C). The results indicated that we identified CFSs in another fetal lung fibroblast, IMR-90.

### 3.2. Molecular Characterization of 1p31.1 Fragile Site

**Figure 2:**
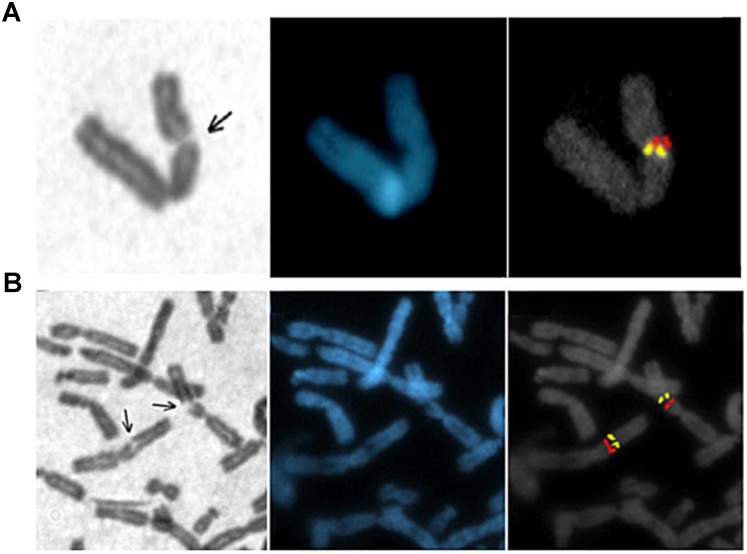
Genomic annotation of 1p31.1 and 3q13.3 fragile regions. (**A**) On the left, break on the short arm of chromosome 1 (1p31.1) was identified with Giemsa staining; in the middle, the same chromosome is visualized with 4′,6′-diamidino-2-phenylindole hydrochloride (DAPI). Distal and proximal probes flanking the common fragile sites (CFSs) are localized, in the same chromosome, through subsequent fluorescent in situ hybridization (FISH) staining (panel on the right). (**B**) On the left, identification of a fragile region on chromosome 3 (3q13.3) using Giemsa staining; in the middle, the same region is visualized with DAPI staining and by FISH of distal and proximal probes flanking the CFS (panel of the right). The arrows show the fragile site location.

The 1p31.1 is located on the short arm of chromosome 1 and spans about 4 Mb (Figure S1 and Table 1) according to the FISH signals obtained using two specific probes for this region (distal RP11-316C12; proximal RP11-297N6), which localized the fragile region in a G-band (Figure 2A).

The Genome Data Viewer NCBI database (build 37) locates 52 different genes along the fragile region (Figure S1), with many of these genes regulating important cellular functions; a long non-coding RNA is also present (*LINC01360*), as well as a microRNA (miR186) known to function as a tumor suppressor in several solid tumors [33]. An 886 kb long gene, *NEGR1* (neuronal growth regulator 1) is also present in the region, underscoring the previously reported association between long genes and CFSs instability [34]. This gene is highly expressed in the brain tissue and is involved in protein metabolism pathways and post-translational modification-synthesis of Glycosylphosphatidylinositol (GPI)-anchored proteins. An important paralog is *LSAMP* (limbic system-associated membrane protein), located in 3q13.3, the other fragile region hereby analyzed. We next performed a DNA repeat composition of the fragile sequence from the fragile region in comparison with non-fragile locus (N-FRA) [31], as well as human genome average (HGA) [32] with similar AT content, to investigate the presence of genetic differences between these regions. The comparisons showed no significant differences between 1p31.1 fragile region, N-FRA, and human genome average (HGA)—a nucleotide-matched genome average with the same 64% AT content—apart from a slight enrichment in Long Interspersed Element-1 (LINE-1), Long Terminal Repeat (LTR), and Mammalian-wide Interspersed Repeats (MIRs) (Figure S2), suggesting that sequence composition is unlikely to be a driving force behind the fragility of the region.

### 3.3. Molecular Characterization of 3q13.3 Fragile Site

The fragile region on the long arm of chromosome 3 spans more than 4 Mb in the 3q13.3 chromosomal region (Figure S3). The probes chosen for FISH analysis (distal RP11-324H4; proximal RP11-305I9) localize at 3q13.3 (Figure 2B) in the proximity of a known G- and a R-band annotated on NCBI. Interestingly, G/R-bands with a higher adenine-thymine guanine-cytosine (AT-GC) interface have been shown to promote genome instability due to a greater difference in corresponding twist angles of DNA [21].

The database analysis revealed the presence of 60 genes, and three of them localize within the boundaries of the fragile region: *LSAMP* (limbic system-associated membrane protein) is at the border of the fragile site closer to the centromere, whereas IGSF11 (immunoglobulin superfamily member 11) and *ARHGAP31* (Rho GTPase activating protein 31) are on the telomeric side of the fragile site, and are, respectively, 1.33, 0.246, and 0.126 Mb long. miRNAs and long non-coding RNAs are also present in this region (Figure S3). *LSAMP*, mapping to the fragile site proximal region, encodes for a member of the immunoglobulin *LAMP*, *OBCAM (Opioid-binding cell adhesion molecule)*, and neurotrimin *IgLON* (Ig-like cell adhesion molecule) family of proteins that contribute to the guidance of developing axons and remodeling of mature circuits in the limbic system. Known to function as a tumor suppressor, its expression is largely tissue-specific and is reported to be high in the brain, bladder, and prostate [35]. 

Next, the analysis of elements characterizing the region was performed with Repeat Masker [30]. For AT content and repetitive elements including LINEs, Alu element, LTRs, and MIRs, and analyzed in comparison with the fragile region, N-FRA, and HGA, finding a nucleotide-matched genome average with the same 63% AT content as the fragile region, showing no significant differences, consistent with our findings for region 1p31.1 (Figure S4).

### 3.4. Presence of Long Genes is a Unifying Feature of CFSs

**Figure 3:**
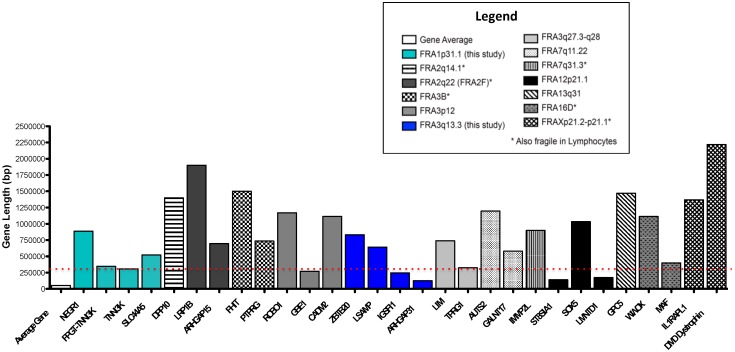
Genes contained within CFSs were found to be longer than the average human gene. Genomic locations of all CFSs experimentally characterized in fibroblasts were defined and all genes mapping to the CFS regions were identified. The gene length is plotted. The plotted average human gene length (first bar) was derived by dividing the sum of the length of all annotated human genes divided by the total gene number. The red line indicates a previously reported > 300 kb cut off for long genes associated with CFSs [13].

Our genomic analysis of 3q13.3 and 1p31.1 fragile sites showed no significant differences in terms of sequence composition, presence of repetitive DNA, and other genomic elements (Figures S2 and S4). Previous work has shown fragile sites hosting large genes [13]. Due to this prior association, we decided to undertake a comprehensive analysis of every gene and coding DNA within CFSs. We pooled all the experimentally characterized CFSs expressed in fibroblasts and in lymphocytes after treatment with APH, and we provided a complete annotation of all genes, excluding the non-coding RNAs found within the span of the fragile regions (Table 1). Comparing the length of genes found within CFSs against the average human gene length [36], we found that every single CFS characterized, including the ones described in this work, contain one or more long gene (Figure 3; Table 1), reiterating the strong correlation between presence of significantly long genes and expression of fragility in both fibroblasts (Figure 3) and lymphocytes (data not shown). We found the majority of genes present within the CFS to be above the previously proposed > 300 kb cut off [13] (red line, Figure 3). Furthermore, our global gene length analysis showed that the two longest genes in the human genome, *CNTNAP2* and dystrophin (*DMD*), are associated with region fragility in lymphocytes, with the latter also expressed in fibroblasts (Figure 3; Table 1). Although long genes have been extensively associated with CFSs, our data indicate that the presence of long genes is a shared and common feature of all CFSs.

**Table 1:** Long genes are found in every CFS in lymphocytes and fibroblast cell lines. Genomic locations of all CFSs experimentally characterized in fibroblasts were defined and all genes mapping to the CFS were identified and catalogued, including CFS name/identifier, mean frequency of expression in percentage, total length of the fragile region, total length of the band as seen in cytogenetic analyses, names of genes present within the CFS, and their size (in Mb). The reference for the molecular and cytogenetic characterization of the specific CFS was also included.

CFS (APH)	Mean Frequency (in %)	CFS Length	CFS Localization in Chromosome Band(s)	Gene(s)	Length	Human Average Gene Length	References
						0.054 Mb	[36]
FRA1B	≈5%–9%	≈8 Mb	1p32.2 51,1-59,7 Mb	1. *DAB1*: DAB adaptor protein 1 (disabled-1) 1. *DAB1*: DAB adaptor protein 1 (disabled-1) (Ensembl)	1. 1,256 Mb 1. 1,552 MB		[37,38]
FRA1p31.1	11.9%–12.6%	≈5.2 Mb	1p31.1 70,8 - 76,0 Mb	1. *NEGR1*: neuronal growth regulator 1 2. *FPGT-TNNI3K: FPGT-TNNI3K* readthrough 3. *TNNI3K*: TNNI3 interacting kinase 4. *SLC44A5:* solute carrier family 44 member 5	1. 0,887 Mb 2. 0,346 Mb 3. 0,309 Mb 4. 0,522 Mb		[14,39], present study.
FRA1E	≈3%–6%	≈3.0 Mbs	1p21.2-p21.3 97,3-100,30 Mb	1. *DPYD*: dihydropyrimidine dehydrogenase gene	1. 0,843 Mbs		[40]
FRA1K	≈2%–3%	≈4.0 Mb	1q25.3-q31.1 185,6-189,5 Mb	1. *HMCN1*: hemicentin 1	1. 0,457 Mb		[39,41]
FRA1H 5 Azacytidine and APH	≈4%–7%	≈11 Mb	1q41-42.1 215,8-226,4 Mb	1. *USH2A*: usherin 2. *ESRRG:* estrogen related receptor gamma 3. *DNAH14*: dynein axonemal heavy chain 14	1. 0,801 Mb 2. 0,634 Mb 3. 0,471 Mb		[42]
FRA2q14.1	3.2%	≈2.0 Mb	2q14.1 114,0-116,5 Mb	*DPP10:* dipeptidyl peptidase like 10	1,4 Mb		[43]
FRA2q14.1 (fibroblasts)	(≈2%–5%)	≈2.0 Mb	2q14.1 114,0-116,5 Mb	*DPP10:* dipeptidyl peptidase like 10	1,4 Mb		[43]
FRA2F	≈3%–4%	≈3.0 Mb	2q22.1-2q22.2 140,2-143,6 Mb	1. *LRP1B*: LDL receptor related protein 1B 2. *ARHGAP15*: Rho GTPase activating protein 15	1. 1,9 Mb 2. 0,697 Mb		[44]
FRA2q22 (FRA2F) (fibroblasts)	4.7%	≈3.4 Mb	2q22 140,2-143,6 Mb	1. *LRP1B:* LDL receptor related protein 1B 2. *ARHGAP15*: Rho GTPase activating protein 15	1. 1,9 Mb 2. 0,697 Mb		[14]
FRA2S	≈4%–6%	> 4.0 Mb	2q22.3–q23.3 147,1-152,0 Mb	1. *MBD5*: methyl-CpG binding domain protein 5 2. *CACNB4*: calcium voltage-gated channel auxiliary subunit beta 4	1. 0,496 Mb 2. 0,266 Mb		[45]
FRA2G	≈10%–15%	> 2.4 Mb	2q24.3–q31 167,5-170,2 Mb	1. *B3GALT1*: beta-1,3-galactosyltra nsferase 1 2. *STK39*: serine/ threonine kinase 39 3. *CERS6*: ceramide synthase 6 4. *MYO3B*: myosin IIIB	1. 0,578 Mb 2. 0, 294 Mb 3. 0,319 Mb 4. 0,477 Mb		[45,46]
FRA2H	≈10%–12%	> 7.0 Mb	2q32.1-q32.2 182,4-189,7 Mb	1. *PDE1A*: phosphodiesterase 1A 2. *ZNF804A*: zinc finger protein 804A 3. *C2orf88:* chromosome 2 open reading frame 88	1. 0,387 Mb 2. 0,341 Mb 3. 0,324 Mb		[45]
FRA3B	≈18%–30%	≈4.3 Mb	3p14.2 59,6-63,9 Mb	1. *FHIT*: fragile histidine triad diadenosine triphosphatase 2. *PTPRG:* protein tyrosine phosphatase receptor type G	1. 1,5 Mb 2. 0,736 Mb		[47]
FRA3p14.2 (FRA3B) (fibroblasts)	1.9%–2.4%	≈4.3 Mb	3p14.2 59,6-63,9 Mb	1. *FHIT*: fragile histidine triad diadenosine triphosphatase 2. *PTPRG*: protein tyrosine phosphatase receptor type G	1. 1,5 Mb 2. 0,736 Mb		[14], present study
FRA3p12 (fibroblasts)	4.0%–7.5%	≈7.5 Mb	FRA3p12 78,5-86,0 Mb	1. *ROBO1*: roundabout guidance receptor 1 2. *GBE1*: 1,4-alpha-glucan branching enzyme 1 3. *CADM2*: cell adhesion molecule 2	1. 1,171 Mb 2. 0,272 Mb 3. 0,1,115 Mb		[14], present study
FRA3q13.3 (fibroblasts)	17.2%–24.3%	≈4.0 Mb	FRA3q13.3 115,6 - 119,7 Mb	1. *ZBTB20*: zinc finger and BTB domain containing 20 2. *LSAMP*: limbic system associated membrane protein 3. *IGSF11*: immunoglobulin superfamily member 11 4. *ARHGAP31*: Rho GTPase activating protein 31	1. 0,833 Mb 2. 0,643 Mb 2. 1,337 Mb (Ensembl) 3. 0,246 Mb 4. 0,126 Mb		[14], present study
FRA3q27.3-q28 (fibroblasts)	4.5%–7.7%	≈2.0 Mb	FRA2q27-q28 188,1-189,9 Mb	1. *LIM*: domain containing preferred translocation partner in lipoma 2. tumor protein p63 regulated 1 3	1. 0,739Mb 2. 0,325 MB		[14], present study
FRA4F	≈3%–4%	≈7.0 Mb	4q22 (4q22.3) 88,2- 94,8 Mb	1. *FAM13A*: family with sequence similarity 13 member A 2. *CCSER1*: coiled-coil serine rich protein 1 3. *GRID2*: glutamate ionotropic receptor delta type subunit 2	1. 0,385 Mb 2. 1,475 Mb 3. 1,507 Mb		[46,48]
FRA6H	≈2%–11%	≈5.0 Mb	6p21.1-p21.2 35,5-40,5 Mb	1. *MAPK14:* mitogen-activated protein kinase 14 2. *BTBD9*: BTB domain containing 9 3. *KIF6*: kinesin family member 6	1. 0,098 Mb 2. 0,472 Mb 3. 0,395 Mb		[49]
FRA6F	≈1%–2%	1.2 Mb	6q21 111,2-112,4 Mb	1. *REV3L:* REV3 like, DNA directed polymerase zeta catalytic subunit 2. *FYN*: proto-oncogene, Src family tyrosine kinase 3. *LAMA4*: laminin subunit alpha 4	1. 0,185 Mb 2. 0,213 Mb 3. 0,147 Mb		[50]
FRA6E	≈12%–24%	≈3.6 Mb	6q26-q27 159,9-163,5 Mb	1. *IGF2:* insulin-like growth factor 2 receptor 2. *PRKN*: parkin RBR E3 ubiquitin protein ligase 3. *PACRG:* parkin coregulated	1. 0,142 Mb 2. 1,38 Mb 3. 0,589 Mb		[51,52]
FRA7B	≈7%–12%	≈12.2 Mb	7p21.3-p22.3 ≈0,5-12,3 Mb	1. *MAD1L1*: sidekick cell adhesion molecule 1 2. *SDK1*: sidekick cell adhesion molecule 1 3. *NXPH1*: neurexophilin 1 4. *THSD7A*: thrombospondin type 1 domain containing 7A	1. 0,471 Mb 2. 0,968 Mb 3. 0,319 Mb 4. 0,462 Mb		[27]
FRA7q11.22 (fibroblasts)	2.8%–7.3%	≈3.7 Mb	FRA7q11.22 67,7 - 71,4 MB	1. *AUTS2*: activator of transcription and developmental regulator AUTS2 2. *GALNT17*: polypeptide N-acetylgalactosaminyltransferase 17	1. 1,195 Mb 2. 0,581 Mb		[14], present study
FRA7E	≈3%–6%	≈4.5 Mb	7q21.11 80,8-85,3 Mb	1. *SEMA3C*: semaphorin 3C 2. *CACNA2D1*: calcium voltage-gated channel auxiliary subunit alpha2delta 1 3. *PCLO:* piccolo presynaptic cytomatrix protein 4. *SEMA3E*: semaphorin 3E 5. *SEMA3A*: semaphorin 3A	1. 0,180 Mb 2. 0,498 Mb 3. 0,410 Mb 4. 0,286 Mb 5. 0,559 Mb		[22]
FRA7K	≈4%–8%	≈0.800 Mb	7q31.1 110,8-111,6 Mb	*IMMP2L*: inner mitochondrial membrane peptidase subunit 2	0,899 Mb		[53]
FRA7q31.1 (FRA7K) (fibroblasts)	2.9%	≈0.800 Mb	7q31.1 110,8-111,6 Mb	*IMMP2L*: inner mitochondrial membrane peptidase subunit 2	0,899 Mb		[14]
FRA7G	≈2%–3%	≈5.0 Mb	7q31.2 112,0-117,0 Mb	1. *DOCK4*: dedicator of cytokinesis 4 2. *FOXP2*: forkhead box P2 3. *MET*: MET proto-oncogene, receptor tyrosine kinase 4. *ST7:* suppression of tumorigenicity 7	1. 0,480 Mb 2. 0,607 Mb 3. 0,126 Mb 4. 0,277 Mb		[54,55]
FRA7H	≈3%–6%	> 2.0 Mb	7q32.3 130,5-133,8 Mb	1. *COPG2*: COPI coat complex subunit gamma 2 2. *MKLN1*: muskelin 1 3. *PLXNA4*: plexin A4 4. *CHCHD3*: coiled-coil-helix-coiled-coil-helix domain containing 3 5. *EXOC4*: exocyst complex component 4	1. 0,163 Mb 2. 0.386 Mb 3. 0,525 Mb 4. 0,297 Mb 5. 0,814 Mb		[20]
FRA7I	≈2%–4%	> 2,0 Mb	7q35-q36.1 144,6-146,8 Mb	1. *TPK1:* thiamin pyrophosphokinase 1 2. *CNTNAP2*: contactin associated protein 2	1. 0,384 Mb 2. 2,304 Mb		[56]
FRA8C	≈4%–7%	≈4 Mb	8q24.13-q21 125,7-129,3 Mb	1. *PCAT1*: (lncRNA) prostate cancer associated transcript 1	1. 0,863 Mb (Ensembl)		[42]
FRA9G	≈1%–3%	≈0.400 Mb	9p22.2 17,1-17,5 Mb	*CNTLN:* centlein	0,375 Mb		[57]
FRA9E	≈2%–5%	≈10 Mb	9q32.1-q33.1 109,0-119,0 Mb	1. *PALM2-AKAP2* fusion 2. *DELEC1*: deleted in esophageal cancer 1 3. *PAPPA*: pappalysin 1 4. *ASTN2*: astrotactin 2	1. 0,532 Mb 2, 0,261 Mb 3. 0.249 Mb 4. 0,992 Mb		[58]
FRA10D	≈4%–6%	≈1.8 Mb	10q21.3-q22.1 65,9-67,7 Mb	*CTNNA3*: catenin alpha 3[65,912,518..67,696,217 (-)]	1,784 Mb		[45]
FRA11E	≈3%–5%	> 3 Mb	11p13 31,2 -35,5 Mb	1. *DCDC1*: doublecortin domain containing 1 2. *KIAA1549L*(ike): also known as C11orf41	1. 0,506 Mb 2. 0,298 Mb		[59]
FRA11H	≈2%–3%	≈8 Mb	11q13.2-q13.4 66,3-74,1 Mb	*SHANK2*: SH3 and multiple ankyrin repeat domains 2	0,757 Mb		[60]
FRA11F	≈4%–5%	≈7.5 Mb	11q14.1-q43 84,2-92 Mb	1. *DLG2*: discs large MAGUK scaffold protein 2. kinase (MAGUK) family 2. *GRM5*: glutamate metabotropic receptor 5 3. *DISC1FP1*: DISC1 fusion partner 1 4. *FAT3*: FAT atypical cadherin 3	1. 2,173 Mb 2. 0,561 Mb 3. 0,664 Mb 4. 0,672 Mb		[61]
FRA11G	≈2%–3%	≈4.5 Mb	11q23.3 113,2-118,5 Mb	1. *NCAM1*:neural cell adhesion molecule 1 2. *NXPE2*: neurexophilin and PC-esterase domain family member 2 3. *CADM1*: cell adhesion molecule 1 4. *SIK3*: SIK family kinase 3 5. *DSCAML1*: DS cell adhesion molecule like 1	1. 0,317 Mb 2. 0,349 Mb 3. 0,335 MB 4. 0,255 MB 5. 0,390 Mb		[62]
FRA12p12.1 (fibroblasts)	1.8%	≈3,7 Mb	12p12.1 22,0-25,7 Mb	1. *ST8SIA1*: ST8 alpha-N-acetyl-neuraminide alpha-2,8-sialyltransferase 1 2. *SOX5:* SRY-box transcription factor 5 3. *LMNTD1*: lamin tail domain containing 1	1. 0,141 Mb 1. 0,373 Mb (Ensembl) 2. 1,033 Mb 3. 0,173 Mb 3. 0,239 Mb (Ensembl)		[14]
FRA13A	≈4%–8%	> 1.0 Mb	13q13.2-q13.3 34,4-35,7 Mb	*NBEA:* neurobeachin	1. 0,730 Mb		[63]
FRA13E	≈2%–5%	> 3.3 Mb	13q21-q22 72,5-75,9 Mb	1. *PIBF1*: progesterone immunomodulatory binding factor 1 2. *KLF12*: Kruppel like factor 12 3. *TBC1D4*: TBC1 domain family member 4	1. 0,234 Mb 2. 0,482 Mb 3. 0,199 Mb		[50]
FRA13q31 (fibroblasts)	2.3%–4.8%	≈5.0Mb	13q31 87,5 - 92,5 Mb	*GPC5* : glypican 5	1,469 Mb		[14], present study
FRA15A	≈2%–3%	≈1.0 Mb	15q22.2 60,4-61,3 Mb	*RORA*: RAR related orphan receptor A	0,741 Mb		[64]
FRA16D	≈15%–25%	>1.0 Mb	16q23.2 78,0-79,7 Mb	1. *WWOX*: WW domain containing oxidoreductase MAF 2. *MAF*: MAF bZIP transcription factor	1. 1,113 Mb 2. 0,399 Mb		[65]
16q23.2 (FRA16D) (fibroblasts)	4.1%–5.5%	> 1.0 Mb	16q23.2 78,0-79,7 Mb	1. *WWOX*: WW domain containing oxidoreductase MAF 2. MAF: MAF bZIP transcription factor	1. 1,113 Mb 2. 0,399 Mb		[14], present study
FRA18C	≈3%–4%	≈2.0 Mb	18q22.1-q22.2 68,7-70,6 Mb	1. *CCDC102B:* coiled-coil domain containing 102B 2. *DOK6*: docking protein 6 3. *RTTN*: rotatin	1. 0,499 Mb 2. 0,448 Mb 3. 0,203 Mb		[66]
FRA22B	≈3%–5%	≈1.8 Mb	22q12.2-q12-3 32,5-33,8 Mb	1. *SYN3*: synapsin III [-] 2. *LARGE*: LARGE xylosyl and glucuronyltransferase 1	1. 0.551 Mb 2. 0,761 Mb		[45]
FRAXB	≈8%–14%	> 1.0 Mb	Xp22.3 6,8-8,1 Mb	1. *PUDP*: pseudouridine 5’-phosphatase 2. *STS*: steroid sulfatase	1. 0,379 Mb (NCBI) 1. 0,480 Mb (Ensembl) 2. 0,208 Mb (NCBI) 2. 0,657 Mb (Ensembl)		[67]
FRAXp21.2-p21.1	≈2%–3%	≈5 Mb	Xp21.2-p21.1 28,5-33,50 Mb	1. *IL1RAPL1*: interleukin 1 receptor accessory protein like 1 2. *DMD:* dystrophin	1. 1,369 Mb 2. 2,220 Mb		[68,69,70]
FRAXp21.2-p21.1 (fibroblasts)	1.5%–7.0%	≈5 Mb	Xp21.2-p21.1 28,5-33,50 Mb	1. *IL1RAPL1*: interleukin 1 receptor accessory protein like 1 2. *DMD*: dystrophin	1. 1,369 Mb 2. 2,220 Mb		[14,70], present study
**Homo sapiens: GRCh38.p13 (GCF_000001405.39)**			Annotation release: 109 release date: 2019-06-14		GRCH38.P13 (GENOME REFERENCE CONSORTIUM HUMAN BUILD 38), INSDC ASSEMBLY GCA_000001405.28, DEC 2013		

Fibroblast CFSs: blue; lymphocyte CFSs: black.

### 3.5. Replication Timing analysis of Fragile Regions

Another unifying feature of CFSs is aberrant replication. Here, we characterized CFS replication timing in asynchronous interphase nuclei analyzed by combining FISH experiments with immunofluorescence against BrdU to identify actively replicating cells. FISH probes were specifically designed to delimitate CFSs edges—one proximal to the centromere and the other distal from the centromere. 

**Figure 4:**
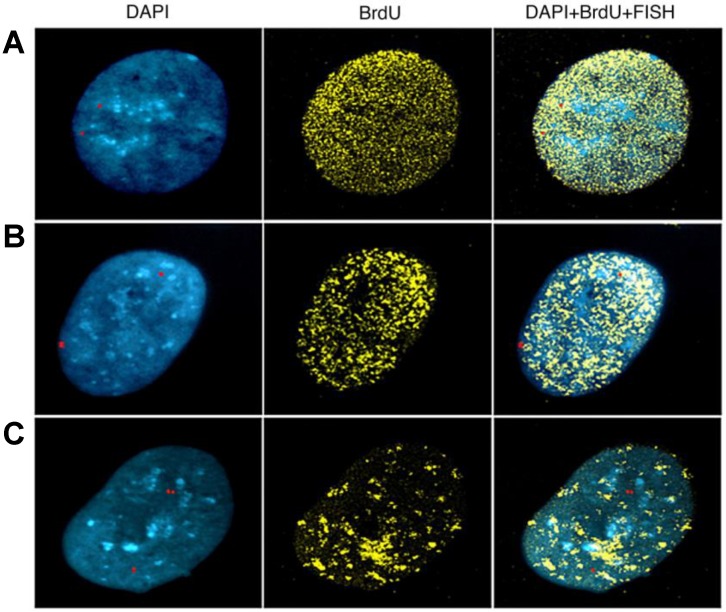
Bromodeoxyuridine (BrdU) replication labelling on interphase nuclei from early, mid, and late S-phases. Example of nuclei stained with DAPI, FISH (red), and BrdU as indicated, and merge (right) of early (**A**), mid (**B**), and late S-phase stages (**C**), as plotted in Figure 5 and Figure 6.

Three temporal stages were identified and classified using the pattern of BrdU incorporation in cycling cells (Figure 4): early, mid, and late S-phase. FISH allowed discrimination between non-replicated allele (S, single spot) from replicated one (D, double spot); the asynchronous alleles are visualized as double and single spot (DS) in each chromosome (Figure 4). This method enabled us to characterize replication timing for both 1p31.1 and 3q13.3 CFSs in both IMR-90 and MRC-5 fibroblasts, as well as a control lymphocytes line where the regions do not manifest fragility, both in the presence or absence of APH. In order to build replication trends throughout the course of S-phase, we further classified the BrdU patters into conventional S-phase stages (I-V) (Figure S5). The early, mid, and late-S-phases (Figure 4) corresponded to stage I, stage III, and stage V (Figure S5), respectively.

### 3.6. Replication Timing Analysis of 1p31.1 Fragile Region

**Figure 5:**
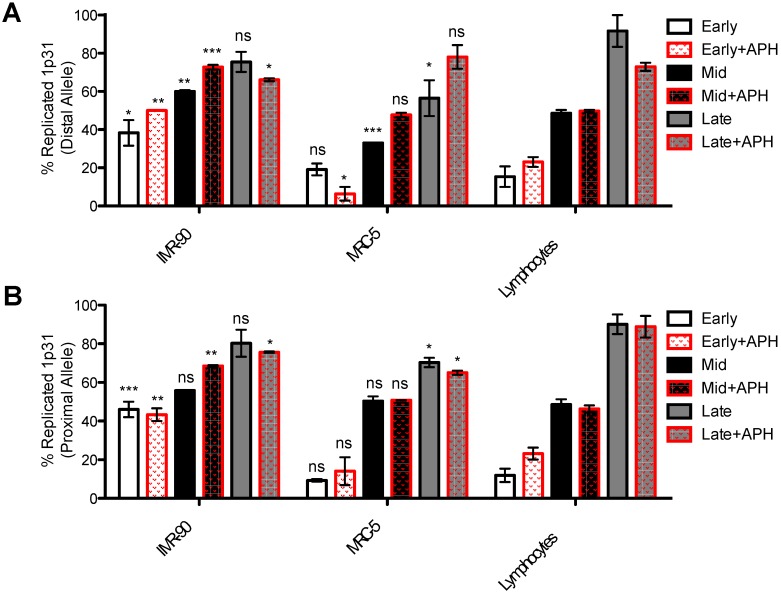
Replication timing analysis of 1p31.1 fragile region on fibroblasts and lymphocytes. The histograms show the percentage of replicated alleles in MRC-5 and IMR-90 cell lines under normal and stressful condition (addition of aphidicolin (APH)). The replication timing of lymphocytes was used as a control. Percentage of replicated distal (**A**) and proximal (**B**) probes are shown. Mean of three individual experiments is plotted, with error bars representing the standard error of the mean (SEM). *p*-values were calculated on the basis of differences between each condition in fibroblasts with the corresponding treatment in lymphocytes. ns: non-significant; * for *p* ≤ 0.05, ** for *p* ≤ 0.01, and *** for *p* ≤ 0.001.

First, we looked at replication timing of the 1p31.3 fragile site using the two probes (proximal RP11-297N6; distal RP11-316C12) in IMR-90, MRC-5, and lymphocytes (Figure 5).

In lymphocytes, where the site is not fragile, replication followed a linear trend, with 50% of alleles found replicated by mid S-phase and 100% of replicated alleles in late S-phase, indicating the expected replication dynamics.

In MRC-5 fibroblasts, the replication of the proximal region followed a wild-type trend through early and mid S-phase that resembled that of lymphocytes, but by the end of S-phase, 25% of alleles were still unreplicated.

We also analyzed for the first time the replication timing in the IMR-90 fibroblast cell line (Figure 5). The IMR-90 cell line shows unique replication dynamics that are distinct from MRC-5 fibroblasts and lymphocytes. Under normal conditions, the trend for replication in this cell line starts in an accelerated fashion. In early and mid phases, the replication of both distal (Figure 5A) and proximal (Figure 5B) alleles was completed rapidly. As previously shown, in different tissues, replication origins can be activated at different times during S-phase [71]; however, our data imply that even within the same tissue, fetal lung fibroblasts; timing of firing; or, alternatively, replisome speed, may be different. Our results are particularly interesting in light of the fact that acceleration in replication fork speed has been recently associated with DNA breaks and genomic instability [72]. Nonetheless, the fact that replication is ultimately completed in unperturbed conditions suggest that faster replication per se does not directly impact region’s stability.

Under conditions of replicative stress using APH, replication dynamics are affected, as expected. Even in lymphocytes, the distal probe arrives in the late S-phase with about 20% of non-replicated alleles, indicating that this region, even if non-fragile in lymphocytes, shows replicative impairments that can be induced by replication stress (Figure 5A). The remaining not replicated alleles are not expressed as breaks, suggesting that they may be replicated and/or repaired in G2 phase [73]. Notably, under APH, both fibroblasts show around 30% of unreplicated alleles (Figure 5). Although it is also possible that replication of both probes may be before mitotic entry, expression of fragility suggests that these significant differences in the number of under-replicated alleles fail to catch up, and unfinished replication directly correlates with CFS expression. 

Next, we attempted to build trends of the replication dynamics throughout S-phase by differentiating BrdU staining into five temporal stages: in addition to early (phase I), mid (phase III), and late (phase V) S-phase (Figure 4), we also picked out cells with BrdU staining likely belonging to phase II (between early and mid) and phase IV (between mid and late) (Figure S5). We applied this qualitative BrdU classification to all three cell lines in both unperturbed and stress conditions and plotted the percentage of replicated alleles for each phase (Figures S6 and S7). In lymphocytes, the replication followed a linear trend, with 52% of alleles found replicated in phase III and 100% of replicated alleles by phase V. The replication trends also confirmed the faster nature of replication of 1p31.1 in IMR-90, which started early in S-phase with proximal and distal probes showing 38% and 25% of D-spots in phase I, respectively, and continued rapidly in phase II (Figure S6). Interestingly, the replication trend in MRC-5 distal probes was initially similar to lymphocytes but became slower, with a stall that lasted from phase II to phase IV, where replicated alleles did not increase (31%, 33%, and 39% in phase II, III, and IV respectively); in phase V, 75% of replicated alleles was seen, similarly to what we showed in Figure 5A,B, and the same percentage was found for the proximal probe.

Under stressful conditions, differences in replication trends between fibroblasts and non-fragile lymphocytes emerged by phases IV and V (Figure S6, right side panels). IMR-90 maintained their faster replication dynamics in early stages in spite of APH treatment, whereas MRC-5 started replication later compared to lymphocytes, suggesting that regulation of processivity under conditions of replication stress may have been operating differently in each fibroblast lines. In MRC-5, the trend suggests that faster replication may be activated between stage III and IV (Figure S6) to promote completion of synthesis of the region in spite of stress, and this event may have had a detrimental impact on the DNA stability. In IMR-90, the proximal probe showed a sharp increase in speed up to phase III (68% D-spots) and then a period of stasis until phase IV, with no more replication happening in phase V. The stasis observed in these late phases could indicate activation of DNA damage responses and underscore fragility of the region, even before replication termination.

Ultimately, incomplete duplication, resolution, or repair of this CFS ahead of mitosis will negatively affect sister chromatids’ resolution and chromosome segregation, resulting in locus fragility. Our data suggest that replication timing, processivity, and paucity may be key features behind this instability. 

### 3.7. Replication Timing Analysis of 3q13.3 Fragile Region

**Figure 6:**
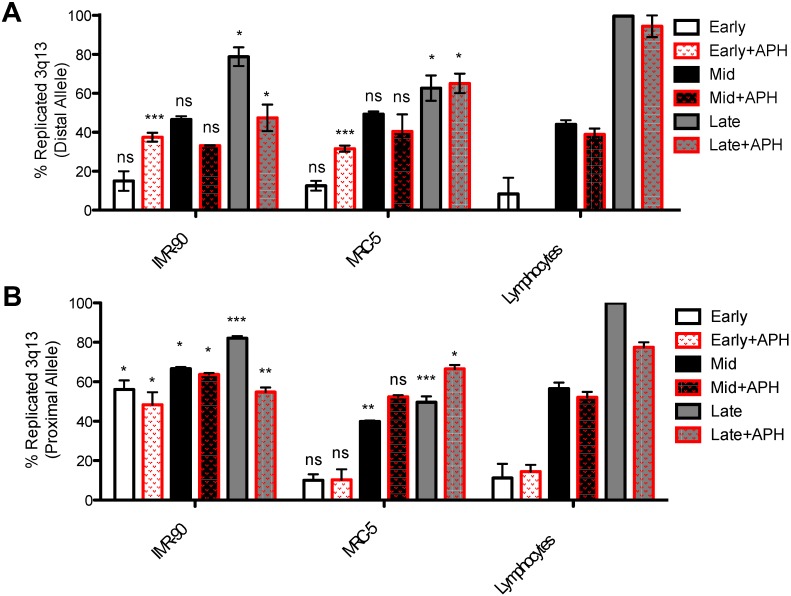
Replication timing analysis of 3q13.3 fragile region on both fibroblast cell lines. The histograms show the replication timing in MRC-5 and IMR-90 cell lines in normal and stressful conditions. The replication timing of lymphocytes was used as control for both fibroblast cell lines. Percentage of replicated distal (**A**) and proximal (**B**) probes are shown. The mean of three individual experiments is plotted, with error bars representing the SEM. *p*-values were calculated on the basis of differences between each condition in fibroblasts with the corresponding treatment in lymphocytes. ns: non-significant; * for *p* ≤ 0.05, ** for *p* ≤ 0.01, and *** for *p* ≤ 0.001.

Replication timing of 3q13.3 was assessed using proximal RP11-324H4 and distal RP11-305I9 probes. In normal conditions, MRC-5 and lymphocytes showed similar dynamics (Figure 6). Both regions started slowly replicating 10%–18% of these alleles in early S-phase (Figure 6A,B). By mid S-phase, only the proximal probe was showing significant difference in MRC-5 replication compared to lymphocytes (Figure 6B).

Importantly, however, 100% of replicated alleles were seen using both probes for lymphocytes, whereas MRC-5 arrived in late S-phase with high proportion of the alleles non-replicated for both the distal and the proximal probes (Figure 6A,B). Our data showed a failure to complete replication in the fibroblast lines, even in absence of aphidicolin, pointing to intrinsic features that affect replication processivity in a cell line-specific way. Given our gene length analysis, and our data showing the ubiquitous nature of long genes within CFS, we suggest that even in unperturbed situations, coordinating replication with transcription and other features of long genes represents a challenge for the cell.

Replication timing of 3q13.3 fragile site in IMR-90 was analyzed here for the first time, showing a very irregular dynamic of replication, with some striking differences between the distal (Figure 6A) and proximal (Figure 6B) alleles that may indicate fork stalling or different paucity along the CFS. Importantly, although the lymphocytes arrived in late S-phase with all of their alleles replicated, IMR-90 failed to replicate 30%–50% of their alleles depending on the probe (Figure 6).

The proximal probe replicated early compared to the distal probe, showing a significantly higher percentage of replicated alleles in early and mid S-phase compared to lymphocytes. Altogether, these data confirm the tissue-specific expression of the 3q13.3 in fetal lung fibroblasts related to intrinsic problems, with replication seen even in unperturbed conditions.

The replication dynamics under APH were more irregular (Figure 6). In MRC-5 and lymphocytes, the proximal probe showed a similar trend in early and mid S-phase (Figure 6B). In lymphocytes, the region arrived at the end of S-phase with close to 100% of replicated alleles for the distal allele in spite of the presence of APH (Figure 6A). Looking at the proximal probe, on the other hand, lymphocytes failed to complete replication of about 20% of the alleles by late S-phase (Figure 6B). Incomplete replication in regions that should be non-fragile is concordant with previous CFS analysis, reporting that these regions are difficult to replicate even in other tissues, suggesting structural impediment for efficient replicative fork progression. Although our analysis uncovered diverse replication dynamics across cell lines and different regions, the common trend was that lymphocytes were able to complete replication within S-phase, whereas CFSs remained under-replicated in fibroblasts.

MRC-5 had an overall similar percentage of replicated alleles as lymphocytes upon APH treatment in early and mid S-phase (with the notable exception of aphidicolin-treated early S-phase, but this was only seen for the distal allele; Figure 6A). Nonetheless, for both alleles, replication failed to be completed by the late phase. 

In IMR-90 fibroblasts, both regions replicated early, starting at around 40%–60% of replicated proximal alleles, after which the replication seemed to stall, terminating with non-replicated alleles at around 25% (Figure 6B); distal alleles, again, replicated early, even in presence of APH, and then encountered a stall that lasted until termination, with the replicated alleles not increasing significantly by late S-phase (50%–60%).

We also built trends of the replication dynamics through S-phase on the basis of BrdU patterns in early (phase I), early to mid (phase II), mid (phase III), mid to late (phase IV), and late (phase V) (Figure S7). The trends of replication were largely linear for all three cell lines. However, replication timing of 3q13.3 fragile site in IMR-90 showed very early replication, with little increase over the course of S-phase, resulting in the late stages IV and V showing incompletely replicated alleles compared to lymphocytes. This was further exacerbated by APH treatment (Figure S7). These data confirmed the results in Figure 6 and suggest that tissue-specific expression of the 3q13.3 in fetal lung fibroblasts likely relates to problems with replication occurring even in unperturbed conditions.

In MRC-5, monitoring trends in replication over the course of S-phase enabled us to detect a stall in replication between phase II and IV (about 54% of replicated alleles in the latter stage) under unperturbed conditions, which was exacerbated under replication stress, with the distal probes showing an irregular trend with delayed replication between phase III and IV (Figure S7). 

Both IMR-90 and MRC-5 cells ended S-phase with a high percentage of non-replicated alleles under unperturbed and APH conditions (Figure S7), as seen in Figure 6. 

Altogether, by end of S-phase, although lymphocytes had completed replication of both alleles, and had replicated about 80% of alleles in APH-treated samples, IMR-90 lagged behind, and this was especially exacerbated under conditions of replication stress, probably not leaving sufficient time for the cell to complete replication prior to mitosis. As per CFS 1p31.1, these results also pointed to intrinsic features of the region that contribute to replication delays. 

## 4. Discussion

In this work, we comprehensively investigated two key aspects of CFSs: replication progression and presence of long genes. Our work confirmed the tissue specificity associated with CFSs for 1p31.1 and 3q13.3 that are expressed in MRC-5 and, as we found, also in IMR-90 fibroblast cells. We quantified the expression of the two fragile regions and found them to be among the most expressed breaks in both fibroblast cell lines and, as previously shown [13], we found no expression of fragility in lymphocytes. 

To confirm the nature of these breakages as CFSs, we showed a percentage of expression higher than 3% and high AT content, typical characteristics of CFSs. The fact that IMR-90 expressed a different fragile locus compared to MRC-5 emphasizes an additional specificity in fragility beyond tissue types, given both cell lines belong to the same tissue. The different frequency of expression for the same CFSs in the two fibroblast cell lines may originate from differences in gender, where MRC-5 is a male cell line and IMR-90 a female one; developmental stage; and also, possibly, by a different transcriptional activity and replication profile in the two cell lines, as we further characterize below. Similarly, these characteristics may affect replication origins and processivity, resulting in earlier replication of these regions in IMR-90 compared to MRC-5, as we have found.

Sequence analysis showed the presence of several genes in both fragile regions, and we found a high majority of them to be above-average in length. For instance, *NEGR1* (0.886 Mb) is located in the core region of 1p31.1, whereas *LSAMP* (1.33 Mb) is found at the boundary of the 3q13.3 core region. It is known that large genes are often late replicating and could promote chromosomal instability on CFSs. Using an online public database, we found a correspondence in both fragile regions between large gene and late replication timing, which could explain the high frequency of expression observed for both CFSs. In 3q13.3 region, we also found the *ARHGAP31* gene (0.126 Mb), an early replicating gene that may promote the fragility by R-loop formation due to replication and transcription machinery collision [25]. 

On the basis of Le Tallec et al. (2013) [13], negligible expression of long genes found in CFS in fibroblast lines was reported, drawing the conclusion that transcription is not associated with fragility of the region. However, monitoring the expression of several of the long genes in the CFSs analyzed here at different time-points during S-phase compared with lymphocytes should give a definite answer whether re-establishment or increase in transcription correlates with fragility of 1p31.1 and 3q13.3. 

We performed a characterization of repetitive and regulative elements to identify additional causes of CFS instability. No differences were found in the percentage of repetitive elements in fragile regions compared to the rest of the genome (Figures S2 and S4), and thus they are unlikely to be major contributors of fragility for 1p31.1 and 3q13.3. 

Knowing that non-canonical replication timing is among the main causes for CFS instability within the genome, we performed replication analysis on MRC-5, IMR-90, and lymphocytes as control to test the hypothesis that expression of fragile sites is correlated with replication impairments. The results showed interesting trends of fragile regions, where IMR-90 consistently showed early replication for both fragile regions, whereas MRC-5 followed a more incremental replication increase throughout S-phase. In a few cases, aphidicolin treatment in MRC-5 caused a modest decrease in the percentage of replicated alleles by late S-phase, which likely reflected temporal discrepancies between experiments. Nonetheless, in both fibroblast lines, the fragile alleles failed to be completely replicated by the end of S-phase in both normal and stressful conditions, suggesting that replication timing per se is not a reason for fragile site expression. Moreover, these regions in lymphocytes, a non-fragile background, also displayed a few instances of non-canonical replication timing and progression, suggesting the presence of intrinsic and structural peculiarities of these regions challenging replicative fork progression. However, in lymphocytes, these regions fully completed replication of both alleles by the end of S-phase, especially under unperturbed conditions, implying that additional mechanisms—perhaps tissue specific transcriptional and epigenetic programs—are final contributors toward chromosomal breakages [71]. Alternatively, or in addition, lymphocytes may have capabilities to complete replication or repair potential damage in G2 phase [74,75,76] that are absent in fibroblasts, where these sites ultimately result in breaks. Although we cannot exclude the fact that replication of these CFS may continue in G2 and/or into mitosis in fibroblasts, delayed replication still has deleterious consequences to genome integrity, as reported for other late replicating regions.

Altogether, our results suggest a prominent role of replication in promoting fragility in these regions, and we put forward the hypothesis that presence of very long genes, whose transcription may be regulated in a tissue-specific and cell cycle stage-specific manner, is a key determinant of replication failure and breakage of the CFSs hereby analyzed. 

The pivotal connection between CFSs and genomic instability was further underscored by presence of genes involved in different diseases that go from tumorigenesis—most of them tumor-suppressor genes—to psychiatric disorders [77,78,79]. 

As essential regions of the genome have been proposed to behave like fragile sites [80], shedding a new light on CFSs’ involvement in promoting genomic instability and in determining the genesis of different types of human disorders is urgently needed. 

## Supplementary Materials

The following are available online at https://www.mdpi.com/2073-4425/11/3/326/s1: Figure S1: Schematic representation of 1p31.3 fragile region. Figure S2: Molecular characterization of 1p31.1 fragile region. Figure S3: Schematic representation of 3q13.3 fragile region. Figure S4: Molecular characterization of 3q13.3.1 fragile region. Figure S5: BrdU replication labelling in interphasic nuclei through stages I, II, III, IV and V, from early to late replication. Figure S6: Replication timing analysis of 3q13.3. fragile region on both fibroblast cell lines. Figure S7: Replication timing analysis of 3q13.3 fragile region on both fibroblast cell lines.
