# Supplementary material for: Impaired Replication Timing Promotes Tissue-Specific Expression of Common Fragile Sites
Source: Genes (Basel). 2020 Mar 19;11(3):326. doi: 10.3390/genes11030326 (PMC7140878; doi:10.3390/genes11030326)
Supplement: Supplementary file 1 [file genes-11-00326-s001.pdf]

genes

MDPI

## Supplementary Materials

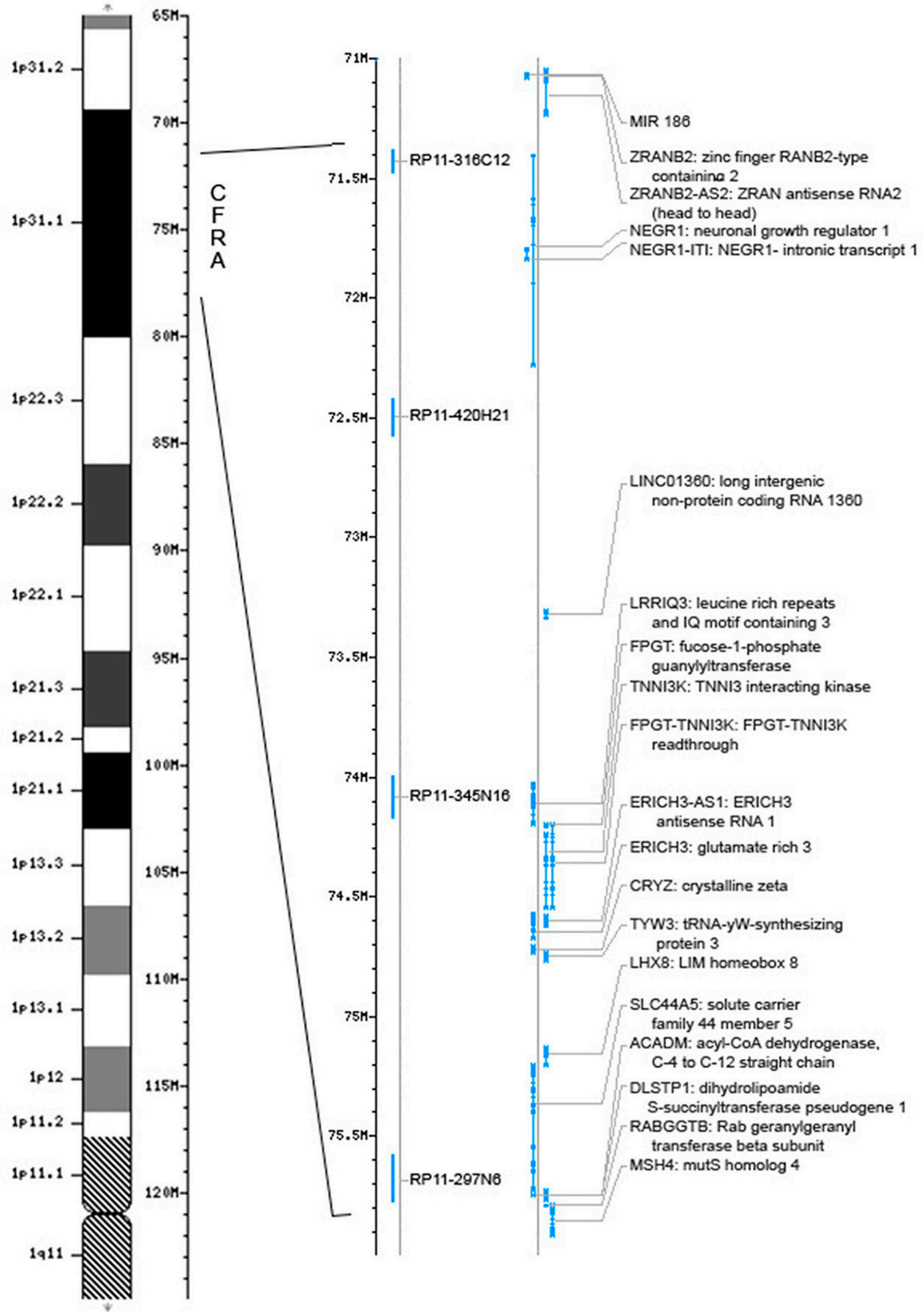


**Figure S1.** Schematic representation of 1p31.3 fragile region. The BAC clones used for the characterization, along with some genes, are shown (adapted from www.ncbi.nlm.nih.gov/mapview).

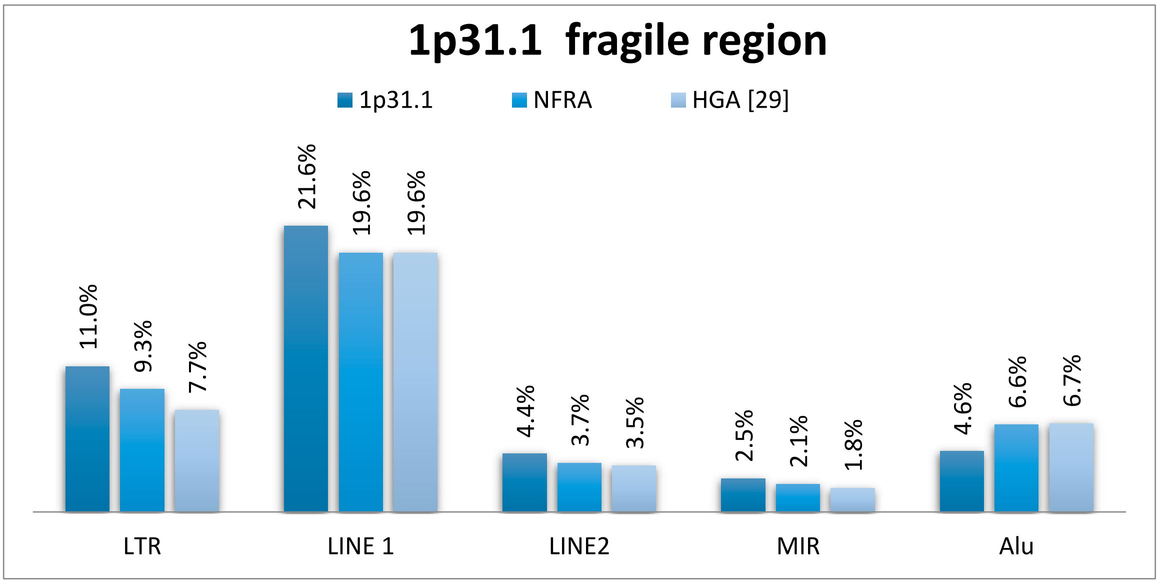


**Figure S2.** Molecular characterization of 1p31.1 fragile region with 64% AT content. Evaluation of A/T enrichment and sequence elements of regions was carried out using NCBI public database (http://www.repeatmasker.org). The analysis was performed on the average of non-fragile regions (N-FRA) and Human Genome average (HGA) with similar A/T content. LINE-1 refers to L1 repetitive elements that are still active retrotransposons in the human genome, while LINE-2 sequences are inactive.

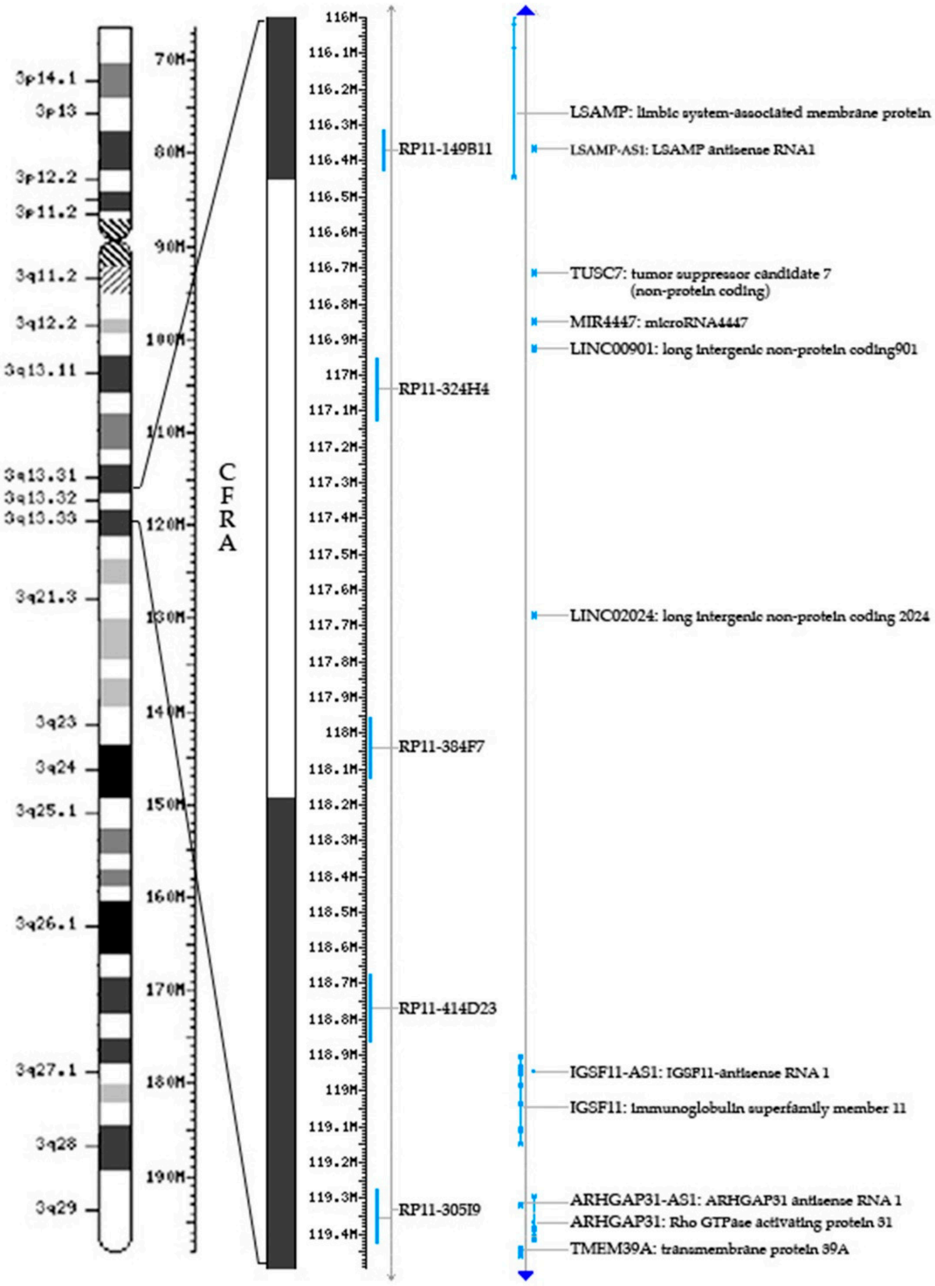


**Figure S3.** Schematic representation of 3q13.3 fragile region; the BACs used for characterization are shown along with some genes (adapted from www.ncbi.nlm.nih.gov/mapview).

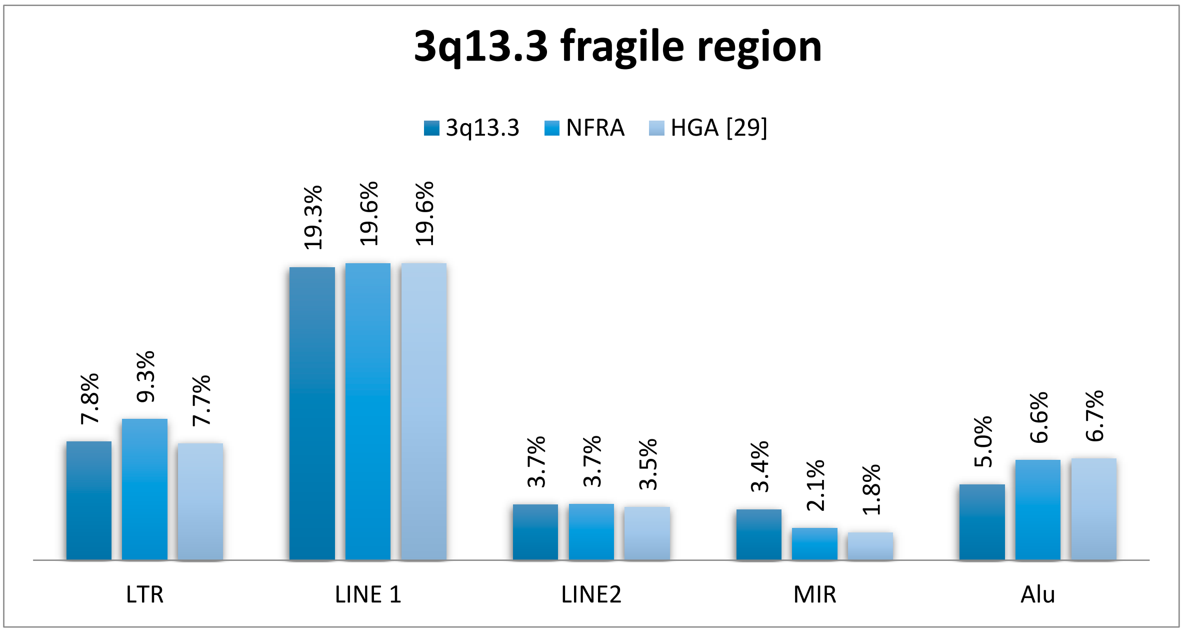


**Figure S4.** Molecular characterization of 3q13.3 fragile region with 63% AT content. Evaluation of A/T enrichment and sequence elements of regions was carried out using NCBI public database (http://www.repeatmasker.org). The analysis was performed on the average of non-fragile regions (NFRA) and Human Genome average (HGA) with similar A/T content. LINE-1 refers to L1 repetitive elements that are still active retrotransposons in the human genome, while LINE-2 repeats are inactive elements.

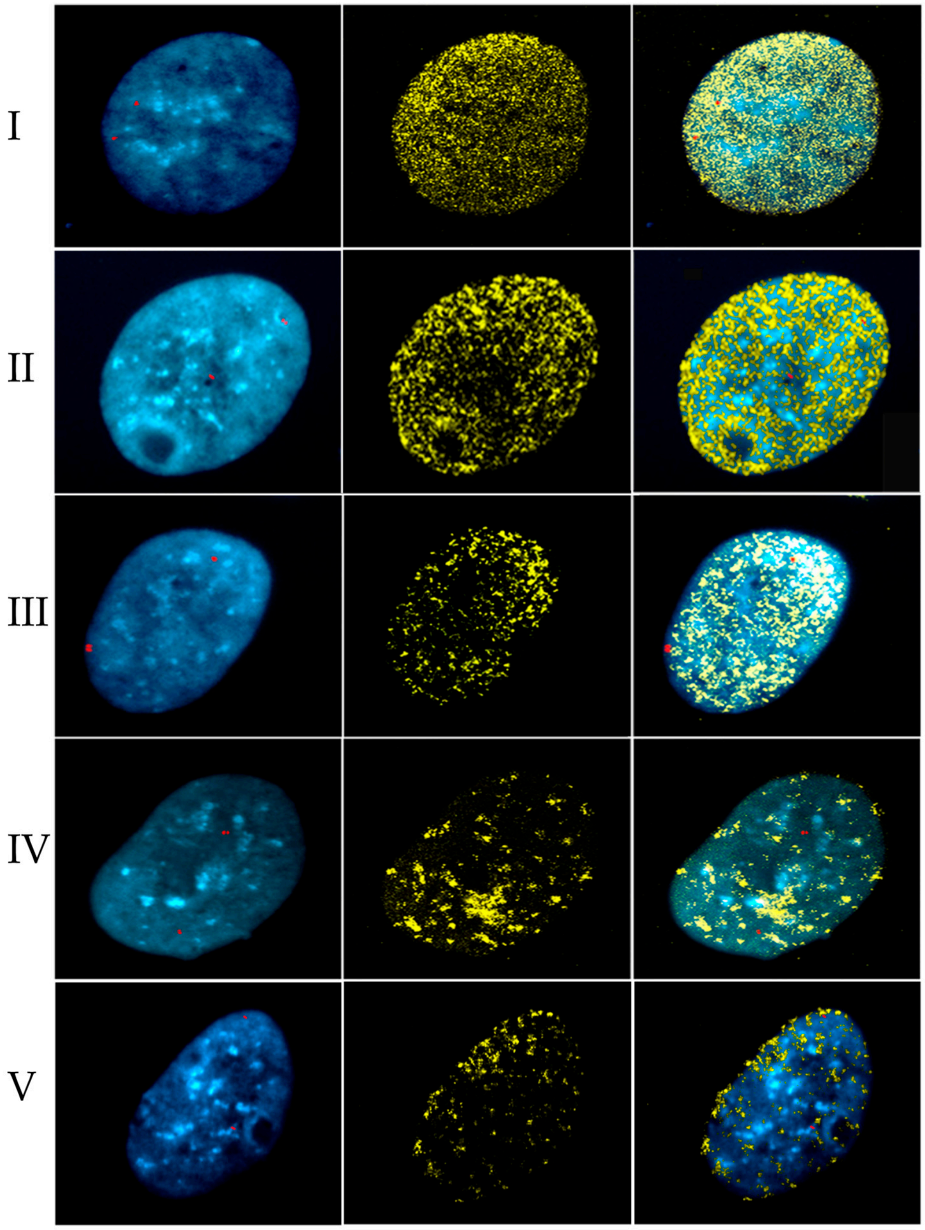


**Figure S5.** BrdU replication labelling on interphasic nuclei through stages I, II, III, IV and V, from early to late replication. Nuclei stained with DAPI, FISH (red), BrdU as indicated, and merge (right) showing S1 phase (I), S2 phase (II), S3 phase (III), S4 (IV) and S5 phase (V).

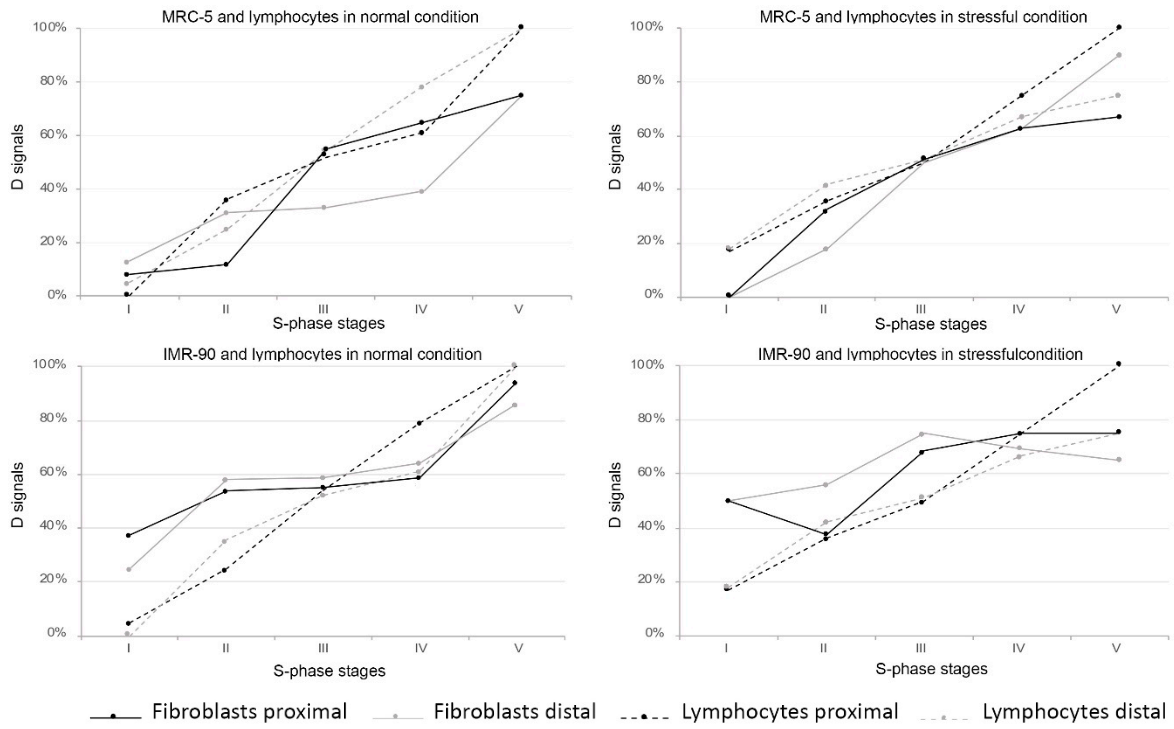


**Figure S6.** Replication timing analysis of 1p31.1 fragile region on both fibroblast cell lines. The graphs show the replication timing trend in MRC-5 cell lines in normal (upper left) and stressful condition (upper right), the replication timing trend of IMR-90 cells in normal (lower left) and stressful condition (lower right). The replication timing of lymphocytes is used as control for both fibroblasts cell lines.

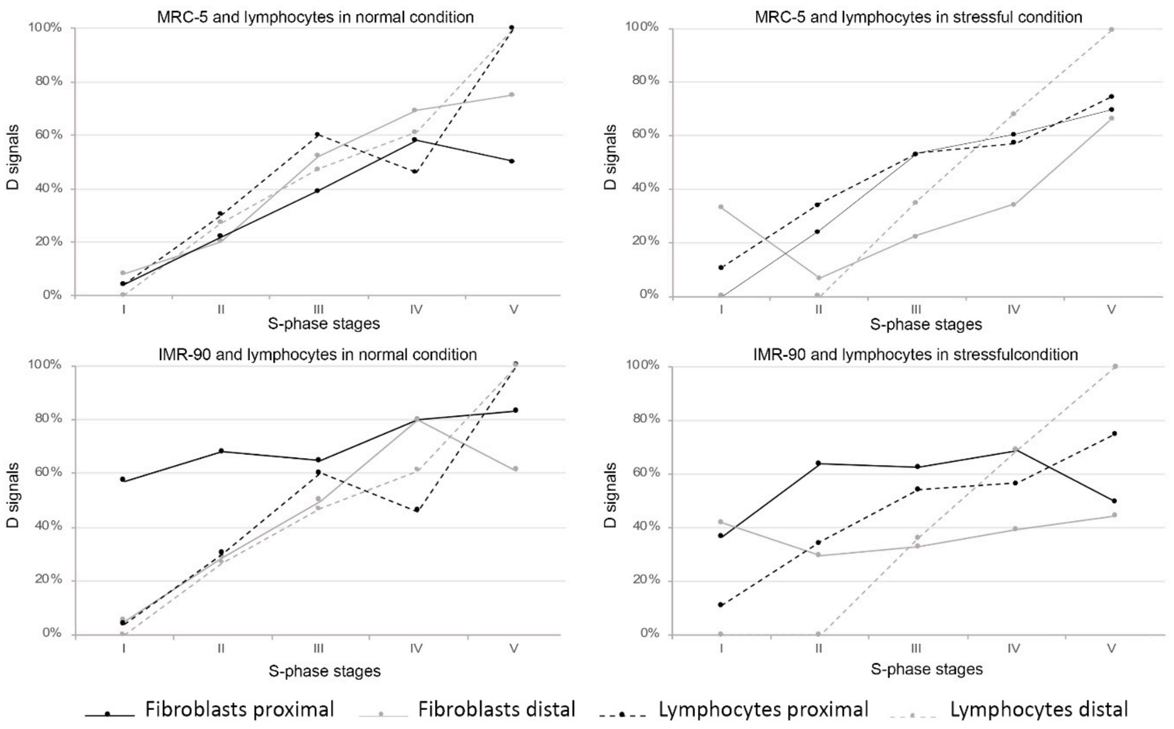


**Figure S7.** Replication timing analysis of 3q13.3 fragile region on both fibroblast cell lines. The graphs show the replication timing trend in MRC-5 cell lines in normal (upper left) and stressful condition (upper right), the replication timing trend of IMR-90 cells in normal (lower left) and stressful condition (lower right). The replication timing of lymphocytes is used as control for both fibroblasts cell lines.

© 2020 by the authors. Submitted for possible open access publication under the terms and conditions of the Creative Commons Attribution (CC BY) license (http://creativecommons.org/licenses/by/4.0/).
